# Salivary Surrogates of Plasma Nitrite and Catecholamines during a 21-Week Training Season in Swimmers

**DOI:** 10.1371/journal.pone.0064043

**Published:** 2013-05-21

**Authors:** Miguel Mauricio Díaz Gómez, Olga Lucia Bocanegra Jaramillo, Renata Roland Teixeira, Foued Salmen Espindola

**Affiliations:** Institute of Genetics and Biochemistry, Federal University of Uberlandia, Uberlandia, Minas Gerais, Brazil; Federal University of São Paulo (UNIFESP), Escola Paulista de Medicina, Brazil

## Abstract

The collection of samples of saliva is noninvasive and straightforward, which turns saliva into an ideal fluid for monitoring the adaptive response to training. Here, we investigated the response of the salivary proteins alpha-amylase (sAA), chromogranin A (sCgA), and the concentration of total protein (sTP) as well as salivary nitrite (sNO_2_) in relation to plasma catecholamines and plasma nitrite (pNO_2_), respectively. The variation in these markers was compared to the intensity and load of training during a 21-week training season in 12 elite swimmers. Overall, the salivary proteins tracked the concentration of plasma adrenaline and were inversely correlated with the training outcomes. No correlations were observed between sNO_2_ and pNO_2_. However, sNO_2_ correlated positively with the intensity and load of training. We argue that the decrease in sympathetic activity is responsible for the decrease in the concentration of proteins throughout the training season. Furthermore, the increase in nitrite is likely to reflect changes in hemodynamics and regulation of vascular tone. The association of the salivary markers with the training outcomes underlines their potential as noninvasive markers of training status in professional athletes.

## Introduction

Periodization is a structured approach based mainly upon the variation of the volume and the intensity of training. Periodization allows athletes to reach maximal performance at appropriate times by providing the necessary physiological adaptation and recovery [1], [2]. Intense and continuous training can induce changes in a broad series of biochemical parameters such as the release of muscle proteins into the blood and variations in cortisol, urea, iron, catecholamines, and blood counts [3], [4]. These parameters are often used to monitor the physiological response to training. Abnormal levels of skeletal muscle proteins in the blood, for instance, can be interpreted as a signal of muscle damage [5]. High concentrations of cortisol and urea are widely regarded as markers of increased protein turnover [6] whereas decreased levels of iron might compromise performance due to its critical role in the delivery and utilization of oxygen by the active muscle [7]. Finally, variations in catecholamines and leukocytes regularly suggest inadequate recovery from training [5], [6]. However, the quantification of these parameters requires blood sampling and it can be inconvenient for the athletes or pose safety risks. Furthermore, for some people venipuncture is painful and stressful. Consequently, the collection of blood might increase the levels of catecholamines and cortisol, thus invalidating the assay. By contrast, the collection of saliva is noninvasive and straightforward. Therefore, analyzing salivary components is clearly appealing in sports medicine.

Catecholamines occupy critical positions in the regulation of physiological processes during exercise. The concentration of plasma catecholamines rises rapidly during exercise, especially at high intensities. This results in increased cardiac output, vasoconstriction in the non-contracting muscles, stimulation of the sweat glands, transportation of oxygen and energetic substrates to the active muscles, and increased contractility of the skeletal muscles [8]. On the other hand, plasma nitrite (pNO_2_) is the product of the oxidation of nitric oxide (NO) and is essential for vasodilation in the systemic and renal vasculature. Further, it has been demonstrated that the concentration of pNO_2_ at rest predicts exercise capacity and is correlated with flow-mediated vasodilation in healthy subjects [9].

Recently, we demonstrated that salivary alpha-amylase (sAA) and salivary nitrite (sNO_2_) show a proportional response to the variation of the intensity and load of training [10]. Salivary alpha-amylase is the most abundant enzyme in saliva and has digestive and anti-microbial properties [11]. The reasoning behind the use of sAA to monitor training is that sAA is released into the saliva mainly after sympathetic stimulation and thus, is considered a surrogate marker for catecholamines [12]. Considering the role of nitrite in vasodilation, we proposed that sNO_2_ would show an equivalent response to the intensity of training [10]. However, the levels of nitrite in saliva are substantially higher than in blood due to the reduction of nitrate by oral bacteria [13]. Therefore, the argument that sNO_2_ is correlated to pNO_2_ warrants confirmation.

A series of studies by Chatterton and colleagues in the late 1990 s stimulated considerable interest in sAA as a marker of sympathetic activity [12], [14]. In these studies, it was reported that the levels of sAA increased significantly before parachute jumping [14] and were correlated with plasma noradrenaline (r = .64) and adrenaline (r = .49) after a single bout of exercise [12]. Ever since, a great deal of research has been devoted to investigating changes in the activity of sAA to a broad series of acute exercise protocols. However, none of them investigated further relations between sAA and catecholamines. On the other hand, less attention has been given to sNO_2_. Few articles have reported changes in sNO_2_ after single bouts of exercise [15], [16] but only our previous study has shown the response of sNO_2_, and sAA, to long-term training under resting conditions. In that study, both sAA and sNO_2_ behaved proportionally, although in opposite directions, to the intensity and load of training. While sAA correlated negatively with the parameters of training, sNO_2_ correlated positively. Because they reflect the activity of biological systems pivotal to the adaptation to training, sAA and sNO_2_ could prove to be appealing markers in sports medicine. Further, to be useful, the variation of salivary parameters in response to training should be assessed under resting conditions, as it is done with most of the traditional markers in blood, and that is why longitudinal designs are particularly important.

The current study aimed to extend the previous findings on sAA and sNO_2_ as markers of the intensity and load of training. We assessed 12 professional swimmers throughout 21 weeks of training. In addition to sAA and sNO_2_, salivary chromogranin A (sCgA), and salivary total protein (sTP) were quantified in saliva. These parameters were compared to plasma catecholamines and pNO_2,_. We hypothesized that there would be a strong correlation between salivary proteins and plasma catecholamines and between sNO_2_ and pNO_2_. We expected that the variations in these markers would be equivalent to the oscillation of the intensity and load of training.

## Methods

### Ethics Statement

The subjects were 12 professional swimmers (8 men and 3 women aged 19.3±1.4 years; BMI 24.3±1.7 kg/m^2^; competition experience; 4.6±2.8 years) different from those who participated in our previous study [10]. None of them smoked, had significant medical or oral health history or was taking regular or incidental medication during the study. One week before the beginning of the collection of the samples, the subjects gave their written informed consent. The experimental protocol was approved by the Institutional Review Board of the Federal University of Uberlandia (Protocol CEP/UFU 483/10).

### Design

**Figure 1:**
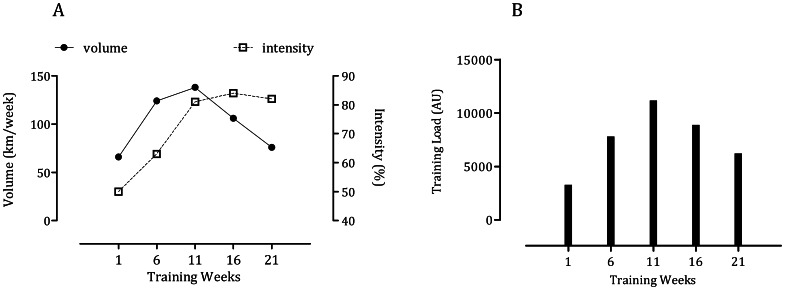
Training outcomes. Figure 1A shows the variation in the intensity and volume of training. Figure 1B illustrates the variation in the load of training (expressed as a function of volume x intensity).

The subjects were evaluated during their regular training season. The experimental design has been described elsewhere [10]. In sum, the subjects completed nine training sessions per week that included predominantly swimming. The volume, intensity and load during the swimming sessions throughout the 5-month study are shown in Figures 1A and 1B. The intensity of training was established by means of blood lactate measures, with an intensity of 100% corresponding to a swimming velocity at the anaerobic threshold for each individual. Every four weeks during the 5-month season, the subjects attended the laboratory under fasting conditions for collection of saliva and blood. All collection procedures took place at 8 am. On each visit, heart rate (HR) and blood pressure (BP) were recorded. The subjects completed the Profile of Mood States Questionnaire (POMS) immediately before the collection of saliva. The POMS is a 65-item questionnaire measuring tension, depression, anger, confusion, vigor and fatigue on a 5-point Likert scale. Diet logs were kept to ensure consistent caloric and nitrate intake for the 48 hours before each sample collection (Table 1).

**Table 1:** Daily dietary intake for the subjects during the 48 hours prior to each collection of the samples.

Total energy (Kcal)	3254 (758.71)
Carbohydrates (g)	486.3 (110.12)
Proteins (g)	143.2 (42.62)
Total fats (g)	84.36 (22.73)
Saturated fats (g)	42.21 (2.97)
Monounsaturated fats (g)	34.53 (2.88)
Polyunsaturated fats (g)	34.78 (5.01)
Nitrate (mg)	84.62 (4.61)

Values are means (SD).

### Sample Collection

Whole saliva was collected with no exogenous stimulation. The saliva was allowed to pool in the mouth and then drooled into pre-weighted collection vials after two minutes. The subjects were asked to refrain from drinking, eating or tooth brushing during the hour prior to the collection of the samples. Immediately after collecting saliva, blood from the antecubital vein (±10 mL) was withdrawn into two EDTA-coated tubes. The samples were obtained by a qualified phlebotomist using standardized venipuncture techniques. The analysis of the blood samples was performed immediately after the collection. The subjects had had their blood routinely sampled prior to this study and none of them reported the procedure as stressful. After the collection of saliva, the samples were stored frozen at −20°C.

### Catecholamines

The blood samples were centrifuged at 4°C for 5 min at 5000 rpm and the plasma was separated. The concentration of plasma catecholamines was determined by high performance liquid chromatography with electrochemical detection. The catecholamines were extracted by alumina and eluted by acetic acid. The separation was performed using a Supelcosil LC-18-DB column (Sigma. St. Louis, MO, USA). The mobile phase consisted of 13.8 g of monosodium phosphate, 100 mg of EDTA, 0.2 g of sodium octanesulfonate, and 30 mL of acetonitrile, pH 3.0, delivered at flow rate of 1 mL/min. The electrochemical detector consisted of a triple-electrode system (Electrochemical Detection, Coulochem, ESA, Chelmsford, MA, USA). The concentration of catecholamines in each sample was corrected using 3,4-dihydroxybenzylamine as the internal standard [17]. The assay sensitivity was approximately 10 pg. The intra-assay coefficients of variation were below 10%.

### Nitrite

To avoid the reaction of nitrite with hemoglobin, the blood samples were immediately centrifuged after collection to separate the plasma. Nitrite was determined by acid diazotation [18]. Fifty µL of saliva or plasma were incubated with 50 µL of Griess reagent (1% sulfanilamide in 2.5% H_3_PO_4_ and 0.1% N-(1-naphthyl)ethylenediamine dihydrochloride) at room temperature for 10 min. The absorbance was measured at 570 nm using a microplate reader (Molecular Devices, Menlo Park, CA, USA). The content of nitrite was calculated based on a standard curve constructed with NaNO_2_ at the concentrations of 400, 200, 100, 50, 25, 12.5, 6.25 and 3.12 µM. The assay sensitivity was approximately 125 pmoL. The intra and inter-assay coefficients of variation for duplicate samples was 4.8% and 6.3% and 12.3% and 14.2% for sNO_2_ and pNO_2_, respectively.

### Salivary Proteins

On the day of analysis, the samples of saliva were thawed and centrifuged at 3000 rpm for 15 minutes. The concentration of the total protein in the samples was determined by the Bradford method [19] using Coomassie Brilliant Blue G-250 (Fisher BioReagents, Fair Lawn, NJ, USA) and bovine serum albumin (Sigma. St. Louis, MO, USA) as the standard protein. The limit of detection of the Bradford method is 1 µg of protein. The intra and inter-assay coefficients of variation for the duplicate samples was 3.2% and 6.1%, respectively. The concentration of the total protein in the samples was used as loading control in western blots. All of the samples from each subject were assayed on the same plate in duplicate. To avoid the possible effects of salivary flow rate on the concentration of proteins, ten micrograms of the total protein from each sample were denatured under reducing conditions and applied on 5–20% SDS–polyacrylamide gradient gels, as previously suggested [20], [21]. The proteins were separated and then transferred onto nitrocellulose membranes in transfer buffer (25 mM Tris, 190 mM glycine, 20% MeOH, pH 7.8–8.4) for two hours at 200 mA and 4°C. The protein transfer was confirmed by visualization with Ponceau. The membranes were blocked for 4 hours at 4°C in blocking buffer (5% non-fat dry milk in PBS w/v). The membranes were then incubated overnight at 4°C with purified polyclonal rabbit anti-human sAA (dilution 1∶5000) (produced in our laboratory) and mouse monoclonal anti-human CgA (dilution 1∶1000) (Millipore, Temecula, CA), respectively. The membranes were subsequently incubated with secondary antibodies for two hours. After the incubations with specific primary and then secondary antibodies, the labeled proteins were detected with ECL reagents and by exposing the developed blots to GE Healthcare films**.** The densitometrical analysis of the spots was performed using ImageJ (U.S. National Institutes of Health, Bethesda, Maryland, USA) by a researcher who was blinded to the experimental design. The area in pixels of each spot was determined in triplicate, and the means were used for statistical analyses.

### Determination of sAA Activity

The samples of saliva were centrifuged at 3000 rpm for 15 min to remove mucins. Ten µL of saliva were diluted (1∶200) in MES buffer (MES 50 mM, NaCl 300 mM, CaCl_2_ 5 mM, KSCN 140 mM, pH 6.3) followed by the addition of 300 µL of pre-heated (37°C) substrate solution (2-chloro-4-nitrophenyl-galactopyranoside maltoside). The optical density was read at 405 nm at one-min intervals during three min at 37°C using a microplate reader (Molecular Devices, Menlo Park, CA, USA). The enzyme activity was determined using the formula: [Absorbance difference per minute × total assay volume (308 ml) × dilution factor (200)]/[millimolar absorptivity of 2-chloro-4-nitrophenyl (12.9) × sample volume (.008 ml) × light path (.97) [10]. The enzyme activity (U/mL) was then multiplied by flow rate (mL/min) to estimate the sAA secretion rate (U/min). The assay sensitivity was approximately 0.4 U/mL. The intra-assay coefficient of variation for duplicate samples was 7.6%.

### Statistical Analysis

The data were tested for normality using the Shapiro-Wilk test prior to the analyses. All of the variables were compared by one-way analysis of variance (ANOVA) with repeated measures followed by the Tukey test for multiple comparisons. The relationships between biochemical markers and training outcomes were analyzed using a two-tailed Pearson correlation coefficient. For all of the analyses, significant results were defined at the level of p<0.05. The results shown are means (SD).

## Results

### Markers of Autonomic Activity

**Figure 2:**
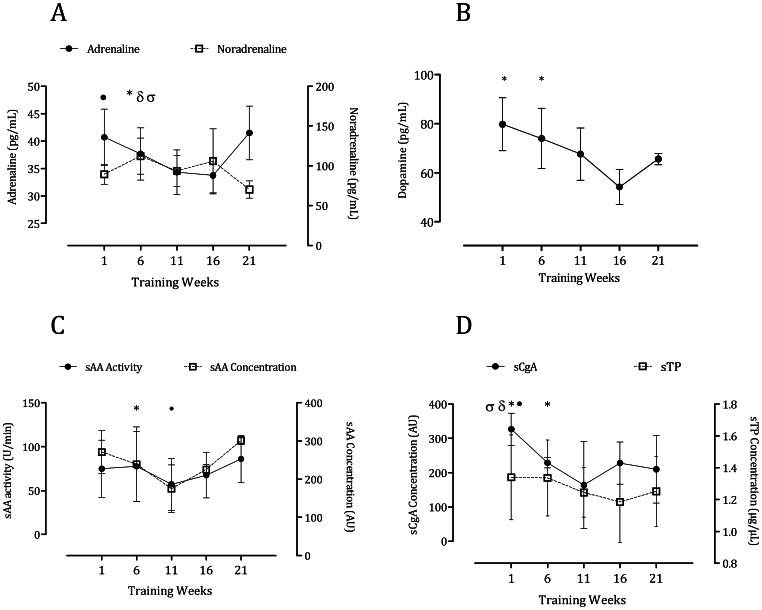
Plasma catecholamines and salivary proteins. Figure 2A shows the variation in adrenaline and noradrenaline in response to training. Adrenaline: • Different from Training week 16. *Different from Training Week 21. Noradrenaline: δ Different from Training Week 11. σ Different from Training Week 21. Figure 2B shows the variation in dopamine. *Different from Training Week 16. Figure 2C shows the response of sAA activity vs. concentration. sAA activity: *Different from Training Week 11. sAA concentration: • Different from Training Week 21. Figure 2D shows the variation in sCgA and sTP in response to training. sTP: *Different from Training Week 11. sCgA: • Different from training week 6. δ Different from training week 11. σ Different from Training Week 16.

**Figure 3:**
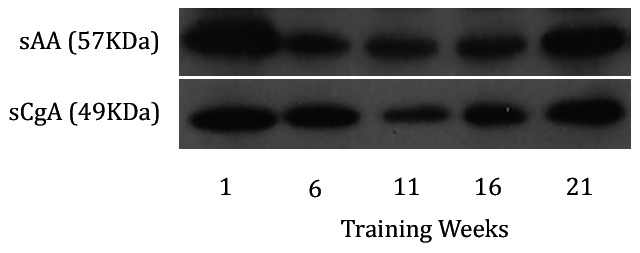
Representative blots of sAA and sCgA in response to training.

**Figure 4:**
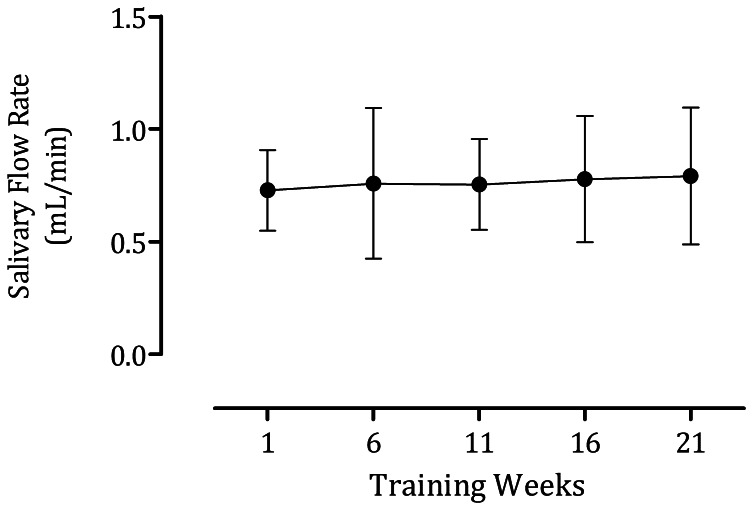
Salivary flow. No difference in salivary flow was observed throughout the training season.

Figures 2A and 2B show the response to training of the markers of autonomic activity. We noted significant differences in adrenaline [*F* (4, 9) = 5.26, p = 0.019], noradrenaline [*F* (4, 9) = 7.47, p = 0.002] and dopamine [*F* (4, 9) = 6.76, p = 0.004]. The concentration of adrenaline and dopamine decreased up to the middle of the training season and subsequently increased towards the baseline levels. On the other hand, noradrenaline oscillated with each month of training. Similar results to those of adrenaline were observed for the salivary surrogate markers. The activity of sAA [*F* (4, 12) = 12.43, p = 0.019], as well as its concentration [*F* (4, 11) = 11.15 p<0.0001], sCgA [*F* (4, 10) = 25.03, p<0.0001], and sTP [*F* (4,9) = 18.24, p<0.001] declined up to the middle of the season with following increases by the end (Figures 2 and 3). No differences in salivary flow rate, HR or BP were observed in response to training (Figure 4 and Table 2).

**Table 2:** Heart rate and blood pressure throughout the 21-week training season.

	Week 1	Week 6	Week 11	Week 16	Week 21
Heart rate (bpm)	64.57 (12.12)	58.20 (6.68)	57.67 (6.32)	57.90 (6.24)	62.83 (9.49)
Systolic pressure (mm Hg)	121.0 (13.70)	109.0 (11.01)	110.0 (9.48)	114.0 (13.50)	116.0 (8.43)
Diastolic pressure (mm Hg)	71.40 (5.70)	67.00 (8.23)	66.50 (6.25)	69.00 (7.37)	67.00 (8.53)

Values are Means (SD).

### Nitrite

**Figure 5:**
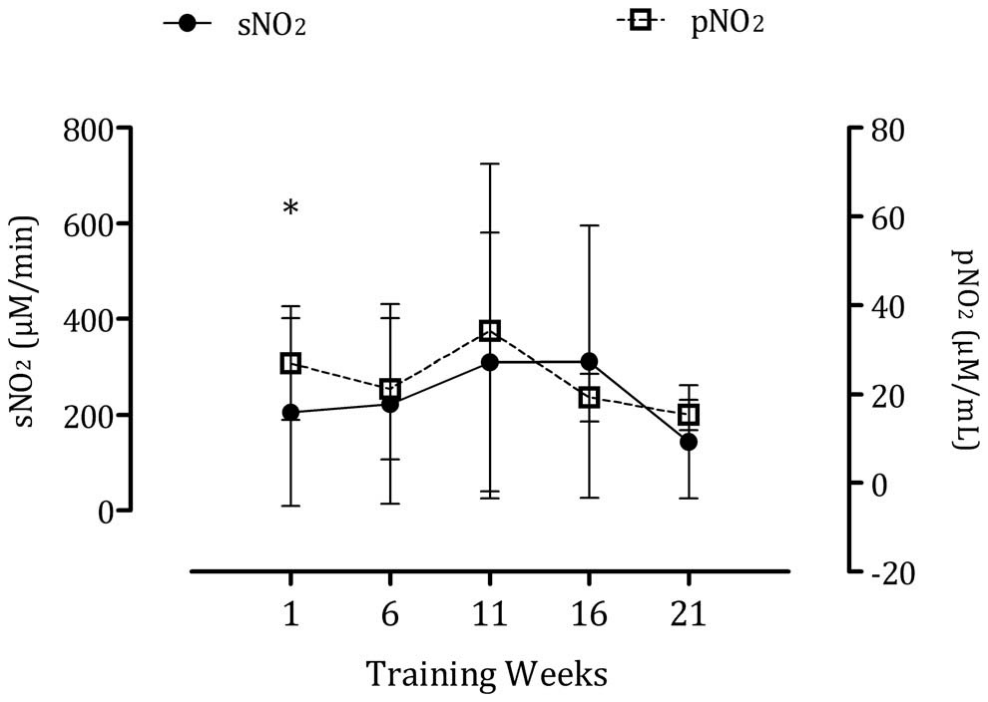
Plasma and salivary nitrite. *Different from Training Week 21 (pNO_2_).

As expected, the concentration of nitrite in saliva was greater than in plasma. The levels of both sNO_2_ [*F* (4, 11) = 3.59 p<0.05] and pNO_2_ [*F* (4, 12) = 6,42 p = 0.027] varied significantly in response to training. However, no significant correlation was found between the two (Figure 5).

### Correlation between Biochemical Markers and Training Outcomes

The Pearson correlation coefficients between the biochemical markers and the training outcomes are shown in Table 3. Throughout the training season, the variation in sAA (both the activity and the concentration) showed a strong positive correlation with adrenaline whereas sCgA and sPT correlated modestly. Only sTP correlated positively with dopamine. We found no significant correlations between the salivary proteins and noradrenaline. Interestingly, we observed a strong positive correlation between the activity and the concentration of sAA [*r*(12) = 0.75, p<0.05]. Furthermore, adrenaline, dopamine and the salivary proteins correlated negatively with the intensity and load of training. On the other hand, sNO_2_ but not pNO_2_ correlated positively with the training outcomes.

**Table 3:** Pearson correlation coefficients between the biochemical markers and the training outcomes at p≤0.05.

	sAA	[sAA]	sCgA	sTP	Adr	Nor	Dop	sNO_2_	pNO_2_
sAA	–	–	–	–	–	–	–	–	–
[sAA]	.75	–	–	–	–	–	–	–	–
sCgA	–	–	–	–	–	–	–	–	–
sTP	–	–	–	–	.78	–	–	–	–
Adr	.83	.89	.52	.59	–	–	–	–	–
Nor	ns	ns	ns	ns	–	–	–	–	–
Dop	ns	ns	ns	.96	–	–	–	–	–
sNO_2_	–	–	–	–	–	–	–	–	–
pNO_2_	–	–	–	–	–	–	–	ns	–
Volume	ns	−.87	ns	ns	ns	ns	ns	ns	ns
Intensity	−.53	−.30	−.85	−.90	−.49	ns	−.88	.33	ns
Load	−.80	−.83	−.46	−.59	−.84	ns	−.57	.68	ns

sAA = salivary alpha-amylase (activity); [sAA] = salivary alpha-amylase (concentration); Adr = adrenaline; Nor = noradrenaline; Dop = dopamine.

## Discussion

Consistent with our original hypothesis, we found strong and modest correlations between the salivary proteins and adrenaline. Further, the salivary proteins and sNO_2_ showed a proportional response to the intensity and load of training. However, contrary to our preliminary hypothesis, sNO_2_ was not associated to pNO_2_ and the latter did not predict the training outcomes.

It is well documented that training modulates autonomic activity. The adaptations associated with long-term training include a decrease in sympathetic activity and an increase in parasympathetic drive [22]. Several reports have found lower resting levels of adrenaline after long-term training in humans [23]–[26]. In these studies, the decrease in adrenaline appears to be more related to the intensity of exercise than to the duration. In our study, catecholamines varied significantly in response to training, and adrenaline was strongly correlated with the load of training.

Although it would be expected that as direct markers of autonomic activity catecholamines, HR and BP displayed similar patterns during training, it is worth mentioning that the autonomic regulation is a tissue-specific and intricate process. Most of the mechanisms linked to the exercise-induced bradycardia, for instance, are thought to be a consequence of increases in vagal tone and a reduction in intrinsic HR. However, a reduction in sympathetic tone is considered to have little effect on the lower HR observed in trained subjects [26], [27]. We failed to observe significant changes in HR and BP. This may be attributed to a “ceiling” effect of training considering the experience of the subjects as competitive athletes, or to the little effect of catecholamines in decreasing sympathetic activity in the heart and blood vessel.

It is interesting to note that the salivary proteins displayed a similar pattern to adrenaline and significantly correlated with the intensity and load of training. As mentioned above, salivary proteins are released into saliva mainly after sympathetic stimulation [28]. Therefore, a decline in the levels of proteins in response to training is expected. Chatterton et al., reported correlations of.64 and.49 between sAA, adrenaline and noradrenaline, respectively, after 10-min intervals of walking, jogging and running [29]. None of the salivary proteins assessed in our study correlated with noradrenaline. Furthermore, although significantly different throughout the training season, noradrenaline failed to correlate with the training outcomes. Under resting conditions, most of the noradrenaline found in plasma is the result of spillover from the sympathetic nerve terminals with a small proportion coming from the adrenal gland [30]. We attribute the lack of correlation between the salivary proteins and noradrenaline in our study to the fact that the subjects were assessed under resting conditions and not after acute submaximal exercise.

Salivary alpha-amylase is the most abundant enzyme in saliva. It is released mainly from the parotid and the submandibular glands and has digestive and anti-fungal properties [11]. Thus far, most of the studies on the sAA response to exercise have incorporated kinetic assays with only a handful of them assessing the concentration of the enzyme [20], [21]. It has been suggested that post-translational modifications such as glycosylations, or the formation of protein complexes between sAA and mucins might affect amylolytic function [31]. Therefore, the concentration of sAA rather than its activity would be a more appropriate marker of autonomic drive. No other study has investigated this association in response to training. Here, we observed a strong correlation between the two (r = 0.75). Additionally, both the concentration and the activity of sAA correlated significantly with the intensity and load of training. It remains to be seen whether other populations, such as type II diabetics or immunocompromised patients show a corresponding response between the activity and the concentration of sAA during exercise.

Salivary chromogranin A is secreted into saliva from the submandibular gland. As a surrogate marker of autonomic activity, sCgA shares the same rationale that sAA. Both are secreted into saliva mainly after sympathetic innervation to the salivary glands. Most studies agree on increments in sCgA in response to exercise. Salivary chromogranin A has been shown to increase proportionally to the intensity of exercise [20], [32] and to correlate with the double product of HR and BP (r = 0.89) as well as the rate of perceived exertion (r = 0.82) during a maximal exercise test [33]. In our study sCgA correlated positively with adrenaline and these two correlated negatively with the intensity and load of training. Our data are in agreement with previous studies showing equivalent responses of sAA and sCgA to acute maximal exercise [20], [21], [32], exposure to microgravity [34] and adverse psychological stimuli [35]. The study that observed a significant increase in sCgA but not sAA in response to exercise did show that both peaked at the end of the exercise and returned to baseline levels 30 min thereafter [33].

Only recently has research identified sTP as a prospective marker in sports medicine. A previous report, for instance, indicates that sTP could predict dehydration after exercise [36]. We have previously proposed that sTP is an ideal marker of autonomic activity [21] and some studies have shown parallel responses between sTP and other salivary proteins during exercise [20], [37]. Here, sTP correlated with adrenaline and the training outcomes. As with sAA and sCgA, the concentration of sTP in saliva rises after innervation to the glands. However, unlike the former proteins, the quantification of sTP is straightforward and inexpensive.

The two major pathways for the production of NO in the body are the arginine/NOS and the nitrate-nitrite-NO pathway. Thus, nitrate and nitrite are important alternative sources of NO and are involved in vascular processes. Nitric oxide is an essential regulator of metabolism and vascular tone. In the striated muscle, NO increases insulin sensitivity, stimulates lipid oxidation and reduces contractility. In the blood vessel, it is known to inhibit contraction, platelet aggregation and promote vasodilation [38]. Here, we have shown that the resting levels of both sNO_2_ and pNO_2_ increase in response to long-term training. However, the variation in one of them does not reflect changes in the other. Dietary nitrate is absorbed in the small intestine and concentrated in the salivary glands. Roughly 5% of the ingested nitrate is reduced to nitrite in the oral cavity by commensal bacteria. Consequently, the levels of nitrate in saliva are approximately 10-fold higher than in plasma [39]. On the other hand, only a fraction of plasma nitrate originates from the NOS pathway [40]. Other groups have presented evidence suggesting that nitrite can be transformed to NO either spontaneously in the blood, or enzymatically in the endothelial cells [41], [42]. Under hypoxic and acidic conditions, such as exercise, nitrite is converted to NO increasing blood flow [43]. This would constitute a NO-scavenging mechanism whereby erythrocytes are unable to form nitrate and methemoglobin. Thus, the levels of sNO_2_ may be more related to plasma nitrate than nitrite. Despite the lack of association between sNO_2_ and pNO_2_, the former did correlate with the training outcomes and constitutes an attractive marker of the load of training in sports medicine.

For a better understanding of the physiological impact of training, we included analysis of glucose, triglycerides, cholesterol, cortisol, urea, iron, lactate dehydrogenase, creatine kinase and complete blood counts (data not shown). These markers were evaluated at the same points than nitrite, catecholamines and the salivary proteins. Additionally, the subjects answered the POMS immediately before the collection of the samples. Glucose, triglycerides, and cholesterol were selected to monitor substantial changes and/or compliance with the diet before each collection point. Because there were no differential changes in any of these parameters and the diet was standardized 48 hours before collecting the samples, we are certain that the variation in catecholamines, salivary proteins and nitrite was due to the oscillation of the training variables. The lack of any significant difference in these parameters during the training season is broadly consistent with several studies in professional athletes which have reported no effect of training in metabolic, hormonal or immunological markers when the periodization of training is appropriate [44]–[47]. It is important to point out that the parameters above have been shown to vary mainly in stages of overreaching and overtraining, which frequently imply grueling loads of training. However, there is no reason to expect any change in these markers when the load of training is properly tolerated. Regarding the female athletes in our study, no difference has been observed in the levels of salivary proteins between men and women under resting conditions [48], [49] or in response to adverse stimuli [50]. Moreover, although we did not control for the phase of the menstrual cycle, it is well documented that the variation of sex hormones during the menstrual cycle does not influence such parameters [51]–[54].

In conclusion, sAA (both the activity and the concentration), sCgA and sTP track the concentration of plasma adrenaline in response to training. Given the simplicity for the quantification of sTP, we propose that sTP be used instead of sAA and sCgA as a marker of autonomic activity. Salivary NO_2_ does not reflect changes in pNO_2_. However, sNO_2_ does predict training outcomes and along with the salivary proteins represent an attractive marker of training status in professional athletes.
